# Sleep and health improvement programme (SHIP) for patients with prostate cancer and caregivers

**DOI:** 10.1002/bco2.435

**Published:** 2024-08-31

**Authors:** Stacy Loeb, Rebecca Robbins, Tatiana Sanchez‐Nolasco, Nataliya Byrne, Andrea Ruan, Adrian Rivera, Natasha Gupta, Stacey A. Kenfield, June M. Chan, Erin L. Van Blarigan, Patricia Carter, Girardin Jean‐Louis, Stephanie L. Orstad

**Affiliations:** ^1^ New York University Grossman School of Medicine New York New York USA; ^2^ Manhattan Veterans Affairs New York New York USA; ^3^ Brigham and Women's Hospital Harvard Medical School Boston Massachusetts USA; ^4^ University of California San Francisco San Francisco California USA; ^5^ UTMB School of Nursing Galveston Texas USA; ^6^ University of Miami Miami Florida USA

**Keywords:** nutrition, physical activity, prostate cancer, sleep, survivorship

## Abstract

**Objective:**

The objective of this study is to determine whether a sleep and health improvement programme (SHIP) to promote healthy sleep, eating and physical activity would be feasible, acceptable and have a positive impact on lifestyle behaviours for prostate cancer survivors and caregivers.

**Methods:**

We recruited 50 participants for a single group 3‐month pre‐post pilot study. The SHIP intervention included (1) website about sleep, nutrition and physical activity (≥1 view/week), (2) two email newsletters with goal‐setting exercises and resources and (3) midpoint health coach call. The primary outcome was changes in validated sleep scales; secondary outcomes included changes in diet, physical activity and concentration from baseline to 3 months.

**Results:**

Of 50 participants enrolled, median age was 65, 30% were Black and 8% were Hispanic. Thirty‐four patients and 7 family caregivers completed the pilot study (82%). Following the intervention, we observed a statistically significant improvement in the Sleep Hygiene Index (pre: 15, post: 13, *p* < 0.01), and a trend toward lower Insomnia Severity Index (pre: 12, post: 9, *p* = 0.07). There were no statistically significant improvements in sleep quality or physical activity, but there were improvements in healthy eating (e.g., increase in cruciferous vegetables and reduction in dairy) and in fatigue‐related problems and concentration. Exit interview feedback was positive.

**Conclusions:**

A web‐based sleep and healthy lifestyle programme for patients with prostate cancer and their caregivers is feasible and acceptable. A randomized controlled trial is planned to test whether a refined SHIP improves sleep and lifestyle in patients with prostate cancer and caregivers.

## 1 INTRODUCTION

Heart disease is a leading cause of death for patients with prostate cancer and is also prevalent among caregivers,^1^, ^2^ who experience high rates of chronic illness, possibly related to de‐prioritization of their own health while caregiving.^3^ Health behaviours, such as good nutrition and regular exercise, are established modifiable factors for managing heart disease risk. Importantly, sleep health has bidirectional relationships with diet and exercise.^4^, ^5^, ^6^ Poor sleep health is pervasive among both patients with prostate cancer and caregivers for cancer survivors.^7^, ^8^ In a survey of patients with prostate cancer, 51% reported poor sleep quality, 18% met criteria for clinical/severe insomnia, and 37% were at high‐risk for sleep apnea.^9^ Also, sleep complaints are a common problem discussed in patient communities, particularly among patients with advanced prostate cancer.^10^ In addition, in a survey of caregivers of patients with prostate cancer, 77% met criteria for poor quality sleep, 26% met clinical criteria for insomnia, 22% had poor sleep hygiene habits, and 43% reported using sleep medications one or more times weekly.^8^ Therefore, modification of sleep habits, healthy eating and physical activity behaviours has the potential to improve cardiovascular disease risk and overall survival, as well as quality of life for patients with prostate cancer and caregivers.^11^, ^12^, ^13^

Numerous interventional studies have been published evaluating healthy eating and/or physical activity programmes in patients with prostate cancer at different stages of disease progression.^14^, ^15^, ^16^ Only in some of these studies was sleep measured among participants. For example, among patients with metastatic castration‐resistant prostate cancer participating in a 12‐week remotely monitored exercise programme, patients in the aerobic exercise arm experienced some improvements in insomnia.^17^ There are limited behavioural interventions designed specifically to promote sleep health in patients with prostate cancer and/or caregivers.^7^ Interventions to improve caregiver well‐being are considered a priority research area by the National Institutes of Health.^18^

To address this gap, we designed the Sleep and Health Improvement Programme (SHIP), with a primary focus on sleep and a secondary focus on healthy eating and physical activity. Objectives of our study were (1) to assess the feasibility and acceptability of the SHIP in a pilot study of patients with prostate cancer and/or caregivers and (2) to assess preliminary effects of exposure to SHIP for improving sleep, healthy eating and physical activity. We hypothesized that the SHIP intervention would be rated as feasible and acceptable by participants and improve patient and caregiver sleep, healthy eating and physical activity from baseline to post‐intervention.

## 2 PATIENTS AND METHODS

From August 2022 to April 2023, we recruited patients residing in the United States with a diagnosis of prostate cancer and/or family members/partners/caregivers of patients with prostate cancer to participate in a pilot study (target *n* = 50). Prospective participants were screened by the study coordinators to confirm eligibility.

**FIGURE 1:**
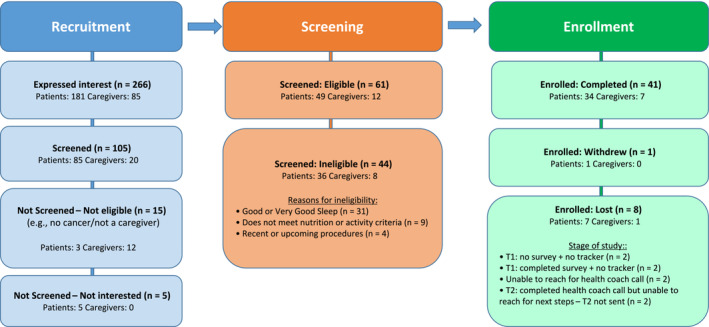
Flow chart for the sleep and health improvement programme (SHIP) pilot study. T1 refers to baseline measures, and T2 is the 3‐month assessment. Of 266 who expressed interest, 125 were able to be successfully reached by phone for screening before the target enrollment was reached. Patients were considered enrolled after signing the consent form; however, two did not complete T1 and could not be reached after consenting. Patients who did not complete the coaching call could not be reached for T2 despite multiple phone and email attempts.

Eligibility criteria were a US adult male with prostate cancer, or a US adult age >18 who is a family member/partner/caregiver of a patient with prostate cancer. There were no limitations on stage or treatment types for prostate cancer. The inclusion criteria were intentionally broad as the SHIP was designed to apply to survivors at different stages of the disease and treatment trajectory as well as family members of patients. Both patients and caregivers were eligible but were not required to join in dyads (i.e., patients did not have to register for partners or family members to participate and vice versa). Telephone and Internet access were required for participants as well as ability to read, comprehend and sign informed consent in English. In addition, participants were screened in advance to confirm that they had a perceived deficit in sleep (using a 5‐point Likert scale for the single question ‘How do you rate your sleep?’ wherein responses of good/very good were excluded) plus a deficit in either nutrition or physical activity (see Supporting information for screening questions and Figure 1 for details on exclusions based on the screener). Participants were excluded if they had a recent (within 6 weeks) or upcoming (within 3 months) medical procedure (i.e., with limitations on physical activity that may affect study participation).

### 2.1 Recruitment

Participants were recruited through the nationwide ResearchMatch platform and prostate cancer support groups (Answer Cancer Foundation, Active Surveillance Patients International, Prostate Cancer Foundation, Fans for the Cure, Malecare and ZERO Prostate Cancer/Us TOO). The study team also recruited participants from the Manhattan Veterans Affairs and New York University Langone Health. We attempted to oversample Black and/or Hispanic participants, groups who are underrepresented in prostate cancer research, using filters in ResearchMatch and targeted recruitment flyers.^19^

### 2.2 Intervention

The study intervention included three components: (1) access to a website with information on sleep health, nutrition and physical activity with instructions to view the website one or more times per week of the study; (2) two digital newsletters sent by electronic mail to the participant at 4 and 8 weeks after completing T1, with goal‐setting exercises and resources to help participants meet their goals; and (3) one check‐in call with a health coach at 6 weeks (20–30 min duration). The SHIP website had separate pages for sleep, physical activity and nutrition with text information and videos featuring experts who provided a summary of the key information and tips. The nutrition content was adapted from prior studies of patients with prostate cancer,^16^ while sleep and physical activity content was developed collaboratively with the expertise of the study team. The website also included a specific page for caregivers; a narratives page with videos from patients, partners and family members sharing their experiences with health behaviour modification; and a summary page with recommendations from each section with downloadable materials. Prior to using the website in the pilot study, user testing was performed with three members of the target audience from different racial/ethnic backgrounds. The website was iteratively modified based on their feedback, including the addition of a dedicated page for mindfulness based on participant comments. Participants were instructed to use the website at least once per week for the 3 months of the study to support their goal setting. They were not required to track their progress with goals as part of the study protocol.

A newsletter was emailed to participants after 1 month that included information on setting a SMART (i.e., ‘Specific, Measurable, Achievable, Relevant, and Time‐Bound’) goal to prepare for their 1:1 call with the health coach at 6 weeks. SMART goal‐setting materials were adapted from a prior weight management study.^20^ During the call, the health coach asked the participant to refine their SMART goal and/or set new SMART goals (two goals total per person) related to sleep, physical activity and/or nutrition. The health coach assisted the participant in adjusting their current goals and/or creating new SMART goals, addressing the barriers they were experiencing to change their health behaviour(s) and recommending additional resources. Their goals were recorded in REDCap, and a second newsletter was sent 2 weeks after the call with individually tailored recommendations (based on their goals) from a physician for resources within the website as well as additional external sources (e.g., links to the American Academy of Sleep Medicine, Prostate Cancer Foundation, American College of Sports Medicine and nutritionfacts.org, with content relevant to their individual goals).

### 2.3 Measures

At baseline, all participants completed a survey and were mailed an actigraph GT3X accelerometer to wear on the wrist for 1 week. Access to the website was provided after completion of the baseline measurements. At the end of 3 months, participants were asked to repeat the survey and wear the accelerometer for one additional week. An exit interview was conducted at the conclusion of the study to gather feedback from participants on the study intervention as well as process measures.

The primary outcomes of the study were a change from baseline (T1) to 3 months (T2) in (1) subjective sleep measures using validated questionnaires and (2) objective sleep measures using actigraphy. The questionnaires included the following:

1. the Sleep Hygiene Index (SHI),^21^ a self‐reported measure with 13 items to assess sleep hygiene behaviours. Items are rated from 0 (*never*) to 4 (*always*) with total scores ranging from 0 to 52 (higher scores represents worse sleep hygiene).
2. the Pittsburgh Sleep Quality Index (PSQI),^22^ including 19 self‐rated items that are combined to form seven component scores, each of which has a range of 0 (*no difficulty*) to 3 points (*severe difficulty*). The seven component scores are added to yield a ‘global’ score (range 0–21); higher scores indicate more severe difficulties in all areas.
3. the Insomnia Severity Index (ISI),^23^ which has seven questions using a 5‐point Likert scale (0–4). The total score range is 0–28; higher scores indicate more severe insomnia (0–7 = *no clinically significant insomnia*, 8–14 = *subthreshold insomnia*, 15–21 = *clinical insomnia moderate severity* and 22–28 = *clinical insomnia severe*).

The three objective measures from actigraphy were (1) sleep efficiency, the ratio of total sleep time (total time spent asleep) to time in bed, reported as a percentage (total sleep time / time in bed from the device × 100); (2) number of awakenings after sleep onset; and (3) sleep duration. Cut‐points from the Freedson 1998 Adult algorithm^24^ were used to score the data, which was downloaded from actigraph data files with epochs set at 60 s with an axis of 3. Participants were also instructed to record their daily sleep and wake‐up times in a sleep log during the entire week of wearing the accelerometer, which two reviewers cross‐examined with the device‐generated times for each actigraph assessment. In cases where a participant's device times were either implausible or considerably different from their sleep log times (e.g., if sleep duration was unrealistically long and if accelerometer seemed to record low activity as sleep), reviewers made case‐by‐case decisions on whether to use the actigraph device or sleep log to derive the measures. If a participant did not return a sleep log, data from the actigraph device was left unchanged and used for each measure. The log was used for 14/36 (38.9%) participants with valid actigraph results.

Secondary outcomes of the study included changes in dietary consumption using the Dietary Screener Questionnaire (DSQ) developed by the National Cancer Institute (NCI) to measure food intake in the past month. Specifically, the Self‐Administered Questionnaire: Paper version of the DSQ from the 2009–2010 National Health and Nutrition Examination Survey (NHANES) was programmed into REDCap and administered online.^25^ To estimate intake of dairy cups per day, we adopted a scoring algorithm from the NCI that converts DSQ responses to estimates of dairy intake in cup equivalents per day using linear regression.^26^ Alcoholic standard drinks per day was measured from Item 2 of the Alcohol Use Disorder Identification Test‐Concise (AUDIT‐C) instrument ranging from 0 (*0 drinks*) to 5 (*10 or more drinks*).^27^

Additional secondary outcomes included three measures for physical activity: (1) change in actigraph‐based moderate‐vigorous intensity physical activity (MVPA) minutes per day, (2) change in actigraph‐based step counts per day and (3) change in self‐reported MET‐minutes per day using the International Physical Activity Questionnaire Short‐Form (IPAQ‐SF).^28^ On valid days of actigraph wear,^29^ defined as ≥10 h worn on a given day, activity counts were classified into metabolic equivalencies using cut‐points from the Freedson Adult 1998 algorithm for moderate and vigorous intensities in adults.^24^ This study required at least four valid days of actigraph wear to be included in the actigraph analyses. All participants also completed several validated instruments including the SF‐12, which includes physical and mental components for which higher scores are better,^30^ Checklist of Individual Strength (CIS) for fatigue with scores of 20–140 (lower scores are better for all subscales)^31^ and the Hot Flash Related Daily Interference Scale.^32^ Three additional domains were evaluated in the questionnaires for patients but not for caregivers: (1) Expanded Prostate Cancer Index Composite (EPIC) hormonal domain, (2) International Index of Erectile Function (IIEF) questions for satisfaction with sex life and confidence to keep an erection and (3) International Prostate Symptom Score questions on nocturia and how you would feel spending the rest of your life with your urinary condition.

For continuous measures, medians at T1 versus T2 were compared using the Wilcoxon signed‐rank test. Proportion measures at T1 versus T2 were compared using McNemar's exact test. All statistical tests were two‐sided, and significance was determined if *p* < 0.05. As this was a pilot study/formative research, our focus was on statistical significance, and multiple comparisons were not addressed. Tests were performed using SAS v.9.4 and R v.4.2.2.

Finally, exit interviews were conducted using a semi‐structured interview guide to obtain qualitative feedback from participants on the intervention itself (if they wished to share any feedback on the overall intervention, website, health coach call), as well as any changes to their physical or mental health that they noted throughout study participation. The NYU Grossman School of Medicine IRB approved the study protocol, and it was registered prospectively on clincialtrials.gov (#NCT 05318131).

## 3 RESULTS

Figure 1 shows the study flow diagram. Among 266 patients and caregivers who responded to one of the study advertisements between August 2022 to April 2023, 125 (47.0%) were contacted successfully by phone during open enrollment, of which 105 (84.0%) completed screening. Of those screened, 61 (58.1%) were eligible to participate, and 50 were enrolled as per our pre‐specified enrollment target.

Of the 50 participants enrolled, 46 (92.0%) completed baseline assessments, 43 (86.0%) completed the midpoint goal check‐in call with the health coach, and 41 (82.0%) completed all study procedures. At 12 weeks, 80.5% (33/41) said they visited the website weekly on most weeks.

Table 1 shows the demographics and baseline characteristics of all enrolled participants (*n* = 50) with a median age of 65, 30% were Black, and 8% were Hispanic. Table 1 also shows the demographics and baseline characteristics for the final study population who completed the SHIP and all study procedures (*n* = 41), including 34 patients and 7 caregivers. A total of 26.8% were Black, and 2.4% were Hispanic. A greater proportion of patients had localized (56.1%) versus metastatic (39.0%) disease. At baseline, 31.7% reported using any sleeping medications.

**TABLE 1:** Baseline demographic characteristics of all enrolled participants (*n* = 42 patients and *n* = 8 caregivers except 2 patients who did not complete T1 surveys) and for the 41 participants who completed the study including patients (*n* = 34) and caregivers (*n* = 7).

	All enrolled participants	Participants completing study (*n* = 41)
Age, years (median, range)	65, 21–78	65, 21–76
Race		
White	34 (68.0%)	28 (68.3%)
Black	15 (30.0%)	11 (26.8%)
American Indian/Alaska Native	1 (2.0%)	1 (2.4%)
More than one race	2 (4.0%)	1 (2.4%)
Hispanic	4 (8.0%)	1 (2.4%)
Prostate cancer spread outside prostate^a^		
Yes	21 (43.7%)	16 (39.0%)
No	25 (52.1%)	23 (56.1%)
Don't know	2 (4.2%)	2 (4.9%)
Comorbidities^b^		
Depression	8 (16.7%)	7 (17.1%)
Diabetes	9 (18.8%)	7 (17.1%)
Heart disease	10 (20.8%)	9 (22.0%)
Pre‐existing sleep conditions^b^		
Obstructive sleep apnea	14 (29.2%)	13 (31.7%)
Insomnia	8 (16.7%)	7 (17.1%)
Restless leg syndrome	4 (8.3%)	3 (7.3%)
Narcolepsy	1 (2.1%)	1 (2.4%)
Using sleeping medication^b^	15 (31.3%)	13 (31.7%)
Using pain medication^b^	12 (25.0%)	10 (24.4%)

^a^ Caregiver participants were asked staging details for the patient with prostate cancer.

^b^ Two participants were enrolled (signed consent) but did not complete the T1 survey.

There were statistically significant improvements in SHI scores from T1 to T2 (median 15 at T1 and 13 at T2, *p* < 0.01); however, no statistically significant differences in PSQI or ISI (Table 2). For the primary objective measures from actigraphy, there was a trend toward less awakenings at T2 (from median of 13.0 at T1 to 10.9 at T2, *p* = 0.06), but no statistically significant difference in sleep duration or efficiency.

**TABLE 2:** Baseline (T1) versus 3‐month (T2) comparisons for primary and secondary sleep, physical activity and nutrition measures among prostate cancer patients and caregivers (*N* = 41).

Measure	T1 (median, range)	T2 (median, range)	T1 to T2 change (mean, range)	*p*‐Value^a^
Primary				
Questionnaires				
Sleep Hygiene Index (SHI) score (0–65, lower is better)	15.0 (3.0–37.0)	13.0 (3.0–29.0)	−2.7 (−17.0–7.0)	<0.01
Pittsburgh Sleep Quality Index (PSQI) score (0–21, lower is better)	9.0 (3.0–16.0)	8.0 (2.0–16.0)	−0.7 (−9.0–8.0)	0.18
Insomnia Severity Index (ISI) score (0–28, lower is better)	12.0 (2.0–26.0)	9.0 (1.0–20.0)	−1.6 (−18.0–8.0)	0.07
Actigraphy				
Sleep efficiency/day^b^	89.6% (59.3%–100.0%)	89.5% (48.9%–98.4%)	0.3% (−46.6%–20.8)	0.28
Number of awakenings/day^b^	13.0 (0.0–31.0)	10.9 (2.9–27.6)	−1.7 (−14.9–14.3)	0.06
Sleep duration (min/day)^b^	383.2 (248.3–628.5)	357.0 (221.6–495.6)	−16.1 (−258.4–106.1)	0.45
Secondary				
Questionnaires				
IPAQ‐SF total non‐MET physical activity min/week	330.0 (0.0–1460.0)	345.0 (0.0–1320.0)	−17.0 (−1260.0–960.0)	0.77
IPAQ‐SF total non‐MET physical activity h/week	5.5 (0.0–24.3)	5.8 (0.0–22.0)	−0.3 (−21.0–16.0)	0.81
IPAQ‐SF total MET physical activity min/week	1290.0 (0.0–6148.5)	1537.5 (0.0–5988.0)	34.3 (−4158.0–4800.0)	0.98
IPAQ‐SF total MET physical activity h/week	21.5 (0.0–102.5)	25.6 (0.0–99.8)	0.6 (−69.3–80.0)	0.96
AUDIT‐C alcoholic drinks/day	0.0 (0.0–5.0)	0.0 (0.0–3.0)	−0.1 (−7.0–3.0)	0.53
DSQ dairy cups/day	1.6 (1.0–6.1)	1.3 (0.9–6.4)	−0.2 (−2.7–0.5)	<0.01
DSQ cruciferous vegetable score	4.0 (0.0–7.0)	5.0 (0.0–8.0)	0.5 (−3.0–5.0)	0.03
DSQ salsa score	2.0 (0.0–8.0)	2.0 (0.0–6.0)	0.0 (−4.0–4.0)	0.78
DSQ tomato sauce score	2.0 (0.0–5.0)	2.0 (0.0–5.0)	0.3 (−4.0–3.0)	0.07
DSQ red meat score	4.0 (0.0–8.0)	3.0 (0.0–7.0)	−0.4 (−6.0–2.0)	0.11
DSQ processed meat score	3.0 (0.0–7.0)	2.0 (0.0–8.0)	−0.4 (−6.0–3.0)	0.09
DSQ oils score	4.0 (0.0–7.0)	4.0 (0.0–7.0)	0.1 (−5.0–3.0)	0.51
DSQ peanuts/tree nuts/nut butters score	5.0 (0.0–8.0)	5.0 (0.0–8.0)	0.0 (−7.0–3.0)	0.89
Actigraphy				
Moderate‐vigorous physical activity (MVPA) min/day^b^	110.3 (2.0–217.0)	105.5 (4.6–268.5)	2.7 (−126.4–89.1)	0.79
Step counts/day^b^	8530.1 (1653.0–15 976.0)	7865.4 (2587.0–19 462.0)	228.2 (−6083.7–4285.1)	0.74

Abbreviations: AUDIT‐C, Alcohol Use Disorder Identification Test‐Concise; DSQ, Dietary Screener Questionnaire; IPAQ‐SF, International Physical Activity Questionnaire Short‐Form.

^a^ Wilcoxon signed‐rank test for pairs was used to assess if the population's values at T2 were different from those at T1.

^b^ *N* = 36 (only participants with at least four valid actigraph days were included for these measures).

Participants reported consuming less dairy and more cruciferous vegetables at T2 compared to T1 (Table 2). Other relevant changes in diet were not statistically significant, such as the reduction in processed meat score (*p* = 0.09). The final study population who completed the actigraph tracking was *n* = 36, including 29 patients and 7 caregivers. There were five participants who did not meet the requirement of four valid actigraph days. There were no statistically significant differences in self‐reported physical activity per day, nor in actigraph‐based MVPA or step counts.

As shown in Table 3, there were no statistically significant differences in physical or mental quality of life scores from the SF‐12, hot flash interference, sexual or urinary health measures. However, there was a statistically significant improvement in the CIS overall scores (scale for fatigue‐related problems), as well as in the specific domain for concentration. Participants also reported feeling less depressed (*p* < 0.01).

**TABLE 3:** T1 versus T2 (median, range) comparisons for secondary subjective sleep, nutrition and physical activity measures among patients and caregivers (*N* = 41).

Measure	T1	T2	T1 to T2 change (mean)	*p*‐Value^a^
Short‐Form 12 (SF‐12): Physical Component Score (higher is better)	47.6 (23.5–53.7)	43.7 (24.2–53.2)	−0.7 (−13.4–19.7)	0.48
SF‐12: Mental Component Score	47.3 (24.1–57.1)	47.6 (25.3–59.2)	1.2 (−12.7–15.5)	0.36
Checklist of Individual Strength (CIS) overall score for fatigue‐related problems (20–140; lower is better for all subscales)	76.0 (53.0–115.0)	70.0 (51.0–99.0)	−5.8 (−54.0–25.0)	0.03
CIS subscale: subjective fatigue (8–56)	35.0 (21.0–44.0)	31.0 (21.0–41.0)	−1.7 (−25.0–14.0)	0.36
CIS subscale: concentration problems (5–35)	17.0 (9.0–34.0)	14.0 (8.0–27.0)	−2.4 (−15.0–9.0)	0.02
CIS subscale: decreased motivation (4–28)	13.0 (6.0–24.0)	13.0 (10.0–23.0)	−0.9 (−10.0–6.0)	0.25
CIS subscale: decreased physical activity (3–21)	11.0 (7.0–17.0)	10.0 (6.0–17.0)	−0.8 (−11.0–10.0)	0.16
Resistance exercise days/week	0.0 (0.0–7.0)	2.0 (0.0–7.0)	0.5 (−2.0–7.0)	0.07
Flexibility exercise days/week	1.0 (0.0–7.0)	1.0 (0.0–7.0)	0.4 (−3.0–7.0)	0.34
Mind and body practice days/week	0.0 (0.0–7.0)	0.0 (0.0–7.0)	0.6 (−7.0–7.0)	0.05
Hot flash interferences with sleep during past week	0.0 (0.0–9.0)	0.0 (0.0–10.0)	−0.1 (−4.0–6.0)	0.60
Expanded Prostate Cancer Index Composite (EPIC): how big of a problem over the past 4 weeks: hot flash^a^	1.0 (1.0–5.0)	1.0 (1.0–5.0)	0.0 (−1.0–2.0)	1.00
EPIC breast tenderness^b^	1.0 (1.0–4.0)	1.0 (1.0–5.0)	−0.2 (−3.0–2.0)	0.30
EPIC feeling depressed^b^	2.0 (1.0–5.0)	2.0 (2.0–5.0)	−0.5 (−2.0–1.0)	<0.01
EPIC lack of energy^b^	3.0 (1.0–5.0)	3.0 (1.0–5.0)	−0.2 (−2.0–2.0)	0.24
EPIC change in body weight^b^	2.0 (1.0–5.0)	2.0 (1.0–4.0)	0.0 (−3.0–3.0)	0.88
International Index of Erectile Function (IIEF): satisfaction with overall sex life over last month^b^	2.0 (1.0–5.0)	2.0 (1.0–5.0)	0.3 (−2.0–4.0)	0.35
IIEF: satisfaction with sex life with partner over last month^b^	2.0 (1.0–5.0)	2.0 (1.0–5.0)	0.4 (−2.0–4.0)	0.15
IIEF: confidence to get and keep erection over last month^b^	1.5 (1.0–5.0)	2.0 (1.0–5.0)	0.1 (−1.0–4.0)	0.48
International Prostate Symptom Score (IPSS): how many times per night do you get up to urinate?^b^	3.0 (0.0–4.0)	2.5 (0.0–4.0)	−0.2 (−2.0–3.0)	0.22
IPSS: feelings about spending rest of life with this urinary condition^b^	2.0 (0.0–4.0)	2.0 (0.0–4.0)	−0.3 (−3.0–2.0)	0.10

*Note*: Shading in the table is used to separate items from different questionnaires.

^a^ Wilcoxon signed‐rank test for pairs was used to assess if the population's values at T2 were different from those at T1.

^b^ *N* = 34 (only patients included for these measures).

Although participants reported they felt significantly more knowledgeable about nutrition related to prostate cancer after completing the study (*p* = 0.02), no differences in the responses to most knowledge questions about nutrition were observed (Table S1). There were trends toward more correct responses about consuming whole milk (harmful) and tomatoes (beneficial).

In terms of goals, at 12 weeks, among participants who reported setting a goal, 60.9% (25/41) completed at least 50% of their sleep goal, 63.6% (21/33) completed at least 50% of their physical activity goals, and 56.4% (22/39) completed at least 50% of their healthy eating goals. Reported barriers included uncomfortable outdoor temperatures posing a challenge with physical activity. Facilitators included having a buddy with whom to make lifestyle changes.

As shown in Table 4, there were statistically significant increases over time in the proportion of who set a SMART goal and kept track of their progress for all three domains. Participants also reported increases over time in other actions such as speaking to friends/family about goals for sleep and healthy eating, changing bedroom environment and purchasing and learning to cook with some of the recommended foods from the website (e.g., cooked tomatoes and cruciferous vegetables). Significantly, more participants reported completing a sleep study (i.e., polysomnography in a medical setting, which is discussed in the sleep portion of the website) during the initial portion of the study than in the final 6 weeks.

**TABLE 4:** Comparisons of the proportions of patients and caregivers who reported improving sleep, healthy eating and physical activity behaviours at 6‐week check‐in call with the health coach versus 12‐week exit interview (*N* = 41).

Question	6‐Week call with health coach (*N*, %)	12‐Week exit interview (*N*, %)	Reported improvement at either 6 or 12 weeks (*N*, %)	*p*‐Value^a^
Sleep—In the past 6 weeks, did you do any of the following to improve your sleep?				
Set a SMART goal	8 (19.5%)	35 (85.4%)	36 (87.8%)	<0.01
Kept track of my progress	4 (9.8%)	14 (34.1%)	16 (39.0%)	0.02
Talked with a friend or family member about my goal	6 (14.6%)	27 (65.9%)	28 (68.3%)	<0.01
Changed my bedroom/sleep environment	9 (22.0%)	25 (61.0%)	28 (68.3%)	<0.01
Talked with a doctor or healthcare provider about improving my sleep	9 (22.0%)	12 (29.3%)	18 (43.9%)	0.61
Completed a sleep assessment/sleep study	10 (24.4%)	2 (4.9%)	12 (29.3%)	0.04
Healthy eating—In the past 6 weeks, did you do any of the following to improve your eating?				
Set a SMART goal	2 (4.9%)	30 (73.2%)	30 (73.2%)	<0.01
Kept track of my progress	2 (4.9%)	12 (29.3%)	12 (29.3%)	<0.01
Talked with a friend or family member about my goal	8 (19.5%)	20 (48.8%)	22 (53.7%)	0.01
Purchased some of the recommended foods	11 (26.8%)	21 (51.2%)	22 (53.7%)	0.01
Learned to cook with some of the recommended foods	6 (14.6%)	18 (43.9%)	19 (46.3%)	<0.01
Spoke with a dietitian	3 (7.3%)	2 (4.9%)	4 (9.7%)	1.00
Physical activity—In the past 6 weeks, did you do any of the following to improve your activity?				
Set a SMART goal	5 (12.2%)	25 (61.0%)	25 (61.0%)	<0.01
Kept track of my progress	6 (14.6%)	16 (39.0%)	17 (41.5%)	0.01
Talked with a friend or family member about my goal	9 (22.0%)	16 (39.0%)	20 (48.8%)	0.12
Purchased exercise equipment or activity monitor/app	6 (14.6%)	9 (22.0%)	13 (31.7%)	0.55
Joined a fitness facility or group fitness program	7 (17.1%)	5 (12.2%)	9 (22.0%)	0.68
Talked with a personal trainer or exercise specialist	5 (12.2%)	5 (12.2%)	8 (19.5%)	1.00

^a^ McNemar's exact test used to compare measures at 6‐week coaching call versus 12‐week exit interview.

In exit interviews, 22 (53.7%) participants reported improvements in physical health (e.g., weight loss, blood pressure and reducing medication use), and 24 (58.5%) reported improvements in mental health (e.g., more energy and less depressed). Additionally, 15 (36.6%) participants reported positive changes in their social interactions as a result of participation, whereas only 4 (9.8%) and 1 (2.4%) reported an impact for their urinary or sexual health, respectively. None of the participants reported any adverse events or decrements in quality of life through participation.

Finally, of 41 participants who completed the exit interview, all (100%) reported that they found the intervention acceptable and most would recommend the study to others. Of the 30 participants who provided feedback on the website during exit interviews, 90% was positive, and 10% was negative. Of 32 participants who gave feedback on the health coach call, 87.5% was positive, and 12.5% was negative. The most common criticism was that the website content did not change over time. Some participants also felt that wearing the actigraph device was uncomfortable or they would have preferred that the intervention included an activity monitor with a display to provide feedback on their sleep and activity.

## 4 DISCUSSION

Our findings reveal that a sleep and lifestyle programme is feasible for patients with prostate cancer and their caregivers. Overall, 82% completed all study procedures, which is in line with previous 12‐week studies of behavioural interventions in patients with prostate cancer.^16^ We observed improvements in sleep hygiene, nutrition and concentration in those that completed the study. Other measures, including sleep quality and physical activity, were unchanged from baseline to follow‐up.

This study was undertaken on the basis of prior data suggesting that sleep problems are pervasive among patients with prostate cancer and their caregivers. Poor sleep health is associated with poor mental health (depression and suicidal ideation),^33^ greater healthcare utilization (medical visits and hospitalizations)^34^ and prostate cancer mortality.^35^ Despite its importance, sleep health is poorly managed among prostate cancer survivors. Sleep health is also not addressed in the American Cancer Society Prostate Cancer Survivorship Care Guidelines despite the significant burden and impact of sleep health problems in this population.^36^ Unfortunately, hypnotic sleeping medications are commonly prescribed in the course of cancer care.^37^ Hypnotic use is associated with impaired cognition and memory, increased risk of accidents and falls,^38^ and greater overall mortality.^39^ Use of hypnotics is ill‐advised when behavioural programmes are available. Meanwhile there are limited data on behavioural strategies for sleep health in the prostate cancer population.

Importantly, a diagnosis of prostate cancer represents a ‘teachable moment’ to engage patients in healthy behaviours.^40^ Previous studies have shown the ability of behavioural interventions to result in lasting positive behavioural changes among patients with prostate cancer.^41^, ^42^ However, there are also many challenges with enacting behaviour change in this population.^43^ For example, participants in our study reported numerous barriers outside of the trial, such as injuries and family issues that interfered with lifestyle modification.

It is important to note that for some endpoints, we did not observe a statistically significant change within the 3‐month study period. For example, for physical activity, we may not have seen statistically significant changes because the study population was already physically active at baseline. According to the transtheoretical model, health behaviour change involves progress through six stages (precontemplation, contemplation, preparation, action, maintenance and termination).^44^ Exit interviews revealed that many participants were still in the preparation stage for some health behaviour changes and were making plans to take action soon or were in the early stages of action (e.g., purchasing some of the recommended foods). Longer term follow‐up would be helpful in further evaluating health behaviour change outcomes.

Since this was designed as a pilot study to assess the feasibility of the intervention, limitations of our study include no control group and the small sample size, particularly for caregivers precluding the opportunity to examine meaningful differences in this subgroup. Because enrollment took place on a rolling basis, we did not modify the website during the course of the study, which many participants felt would have improved the quality of the intervention. Additionally, recruitment of caregivers was more challenging since most registries (e.g., ResearchMatch) and clinic‐based recruitment methods focus on patients with a health condition and do not provide a readily accessible method for identifying caregivers. Since caregiver health and well‐being is a priority area for research, registries for caregivers would be extremely helpful to facilitate study recruitment. Additionally, despite the use of targeted flyers and filters in ResearchMatch, few Hispanic participants enrolled in and completed the study.

Strengths of the study are the use of tailored information on the website (e.g., quizzes providing individual scores and information for sleep health) and newsletters for patients with prostate cancer and caregivers. Message tailoring refers to the design of health messages that are unique to characteristics of a particular population, rather than using off‐the‐shelf messaging that does not address specific barriers and facilitators.^45^ Additionally, very few studies have addressed sleep in caregivers,^46^ and to our knowledge, this is the first study to do so for caregivers of patients with prostate cancer. An additional strength of our study is the comprehensive assessment of sleep health through multiple validated questionnaires as well as actigraphy. Only a few studies have previously used actigraphy to measure sleep in patients with prostate cancer,^47^, ^48^ and these studies found a significant discrepancy between self‐reported measures of sleep health and device‐based measures.

## 5 CONCLUSIONS

This study supports the feasibility and acceptability of a sleep and healthy lifestyle programme for a diverse group of patients with prostate cancer and their caregivers. There were preliminary improvements in sleep hygiene, nutrition and concentration following exposure to the SHIP. Feedback from participants suggested the value of evidence updates and more frequent contacts/reminders to motivate ongoing health behaviour modification. A randomized controlled trial is planned to test whether a refined SHIP improves sleep and health behaviour outcomes in patients with prostate cancer and their caregivers.

## AUTHOR CONTRIBUTIONS

Conceptualization (All authors) Data curation (RR, TSN, NB, AR, AR, SLO) Formal analysis (TSN, AR) Funding Acquisition (SL, RR, SLO) Methodology (All authors) Project Administration (SL, TSN, NB) Software (TSN, AR, SLO) Visualization (AR) Writing—Original Draft (SL, AR) Writing—Review and Editing (All authors)

## CONFLICT OF INTEREST STATEMENT

SL declares consulting with Astellas (unrelated to the current study). SAK declared consulting with Fellow Health, Inc (unrelated to the current study).

## Supporting information


**Figure S1.** Screening questions on sleep, nutrition and physical activity.
**Table S1.** Comparisons of proportions of patients and caregivers who answered nutrition knowledge questions correctly in T1 vs. T2 (N = 41).
