# Supplementary material for: Sleep and health improvement programme (SHIP) for patients with prostate cancer and caregivers
Source: BJUI Compass. 2024 Aug 31;5(10):976–85. doi: 10.1002/bco2.435 (PMC11479809; doi:10.1002/bco2.435)
Supplement: Supplementary file 1 — Figure S1. Screening questions on sleep, nutrition and physical activity. Table S1. Comparisons of proportions of patients and caregivers who answered nutrition knowledge questions correctly in T1 vs. T2 (N = 41). [file BCO2-5-976-s001.docx]

**Supplemental Items.**

**Supplemental Figure 1. Screening questions on sleep, nutrition and physical activity.**

Sleep Screener (required to qualify):

○ How do you rate your sleep? (5 point Likert scale: very poor, poor, average, good, very good- exclude if respond good/very good to this question)

AND

Diet and Physical Activity (must have at least 1 area for improvement in either diet or physical activity)

○ Do you consume processed meat or whole milk? Yes/No (If yes, they qualify)

OR

○ Do you consume vegetables every day? Yes/No (If no, they qualify)

OR

○ How often do you do aerobic physical activity (“cardio”)? (If <150mins per week they qualify)

**Supplemental Table 1.** Comparisons of proportions of patients and caregivers who answered nutrition knowledge questions correctly in T1 vs. T2 (N=41)

| **How are the following foods related to prostate cancer?** | **T1 (N, %)** | **T2 (N, %)** | ***P*-value*** |
| --- | --- | --- | --- |
| **Alcohol (neutral)** | 12 (29.3%) | 15 (36.6%) | 0.55 |
| **Bacon (harmful)** | 32 (78.1%) | 35 (85.4%) | 0.51 |
| **Beef steak (harmful)** | 27 (65.9%) | 32 (78.1%) | 0.23 |
| **Cheese (harmful)** | 17 (41.5%) | 23 (56.1%) | 0.18 |
| **Whole milk (harmful)** | 20 (48.8%) | 28 (68.3%) | **0.06** |
| **Broccoli (beneficial)** | 37 (90.2%) | 39 (95.1%) | 0.63 |
| **Tomatoes (beneficial)** | 28 (68.3%) | 35 (85.4%) | **0.07** |
| **Olive oil (beneficial)** | 31 (75.6%) | 30 (73.2%) | 1.00 |

Note: *McNemar’s exact test used to compare measures at T1 vs. T2
